# TRIM21/Ro52 - Roles in Innate Immunity and Autoimmune Disease

**DOI:** 10.3389/fimmu.2021.738473

**Published:** 2021-09-06

**Authors:** Esther L. Jones, Stephen M. Laidlaw, Lynn B. Dustin

**Affiliations:** Kennedy Institute of Rheumatology, Nuffield Department of Orthopaedics, Rheumatology, and Musculoskeletal Sciences, University of Oxford, Oxford, United Kingdom

**Keywords:** innate immunity, E3 ubiquitin ligase, autoimmune disease, Sjogren’s syndrome, Fc receptor, systemic lupus erythematosus, intracellular antibodies

## Abstract

TRIM21 (Ro52/SSA1) is an E3 ubiquitin ligase with key roles in immune host defence, signal transduction, and possibly cell cycle regulation. It is also an autoantibody target in Sjögren’s syndrome, systemic lupus erythematosus, and other rheumatic autoimmune diseases. Here, we summarise the structure and function of this enzyme, its roles in innate immunity, adaptive immunity and cellular homeostasis, the pathogenesis of autoimmunity against TRIM21, and the potential impacts of autoantibodies to this intracellular protein.

## Autoimmune Diseases Associated With TRIM21

The immune system is a balanced network of interacting cells which distinguish between self and non-self to effectively respond to invading pathogens. Failures in these peripheral and central tolerance mechanisms can lead to immune cells reacting to self-antigens, causing the extensive inflammation and tissue damage observed in autoimmune diseases (1).

TRIM21 (also called Ro52) autoantibodies have been detected in at least thirteen autoimmune diseases, with frequencies of detection in patients ranging from 5% to 95% (2, 3). The most common TRIM21-associated autoimmune diseases are systemic lupus erythematosus (SLE) affecting the central nervous system, skin, kidneys and joints, and Sjögren’s syndrome (SS), which primarily affects the tear and salivary glands (4, 5). Beyond SS and SLE, anti-TRIM21 antibodies have also been identified in patients with primary biliary cirrhosis, idiopathic inflammatory myopathies (mainly polymyositis and dermatomyositis), and infants with congenital heart block (CHB) associated with maternal autoantibody transfer (6–9). In fact, for primary Sjögren’s syndrome (pSS), serological detection of anti-TRIM21 antibodies is a diagnostic criterion, with detection rates in patients ranging from 50-70% according to assay method (10, 11).

For TRIM21-associated SS, single nucleotide polymorphism (SNP) and genome-wide association (GWAS) studies have identified polymorphisms in HLA, interferon regulatory factor-5 (IRF5) (suggesting TRIM21 is an interferon-stimulated gene; see below), and B cell activating factor (BAFF) loci (12, 13). For example, it is postulated that specific gene polymorphisms, including a CGGGG indel repeat in the IRF5 gene promoter, can alter IRF5 mRNA expression. This specific repeat may act as a binding site for transcription factor SP1, driving chronic type I interferon (IFN-I) proinflammatory cytokine production (14, 15).

Despite the identification of genetic risk factors (e.g. the influence of SNPs in autoantibody induction) and some of the cellular interactions involved, antigen-specific mechanisms which initiate and drive autoimmune pathologies remain poorly understood. Analysis of peripheral blood mononuclear cells (PBMCs) isolated from SLE and SS patients provides evidence of elevated TRIM21 transcript expression; however, questions remain as to whether TRIM21 is a key autoantigen driving B-cell activation, autoantibody production and autoimmune pathogenesis in these diseases (16).

## TRIM Protein Family

Comprising more than 80 members, the TRIM protein family is a group of E3 ubiquitin ligases with roles in multiple cellular processes including cell cycle regulation, autophagy and innate immunity (17, 18). They have a conserved multidomain architecture, exclusively found in metazoans (both vertebrate and invertebrate species), consisting of the tripartite motif (N-terminal Really Interesting New Gene (RING) domain, B-box domain and coiled-coil domain) that may be associated with a variable C-terminal domain.

Specific C-terminal domains mediate substrate recognition, localisation within the cell and are used to classify TRIM proteins into sub-families according to function (19). This is illustrated by the C-terminal subgroup One Signature domain which enables localisation of TRIM18 (MID1) to microtubules for polyubiquitination and proteasomal degradation of the protein phosphatase 2A catalytic subunit (PP2Ac) (20, 21). Involved in multiple signalling pathways, PP2Ac regulates the mammalian Target of Rapamycin Complex 1 (mTORC1) pathway which controls tumour cell growth and metabolism, intracellular transport, cell migration, autophagy and cell cycle dynamics. When active, TRIM18 polyubiquitinates PP2Ac leading to its degradation, thus inhibiting mTORC1 complex formation to prevent downstream signalling (22). Therefore, it is unsurprising that mutations that alter or inhibit TRIM protein functions have complex downstream effects.

Other family members contain single or combinations of the following C-terminal domains; fibronectin type 3, plant homeodomain, transmembrane, ADP ribosylation factor-like, MATH (meprin and tumour-necrosis factor receptor (TNFR)-associated factor (TRAF) homology), filamin-type immunoglobulin, NHL repeats, or most commonly, a PRY/SPRY domain (23). Given the number of C-terminal domains and proteins in this family, it is conceivable that there is little or no functional redundancy between family members, yet this remains to be confirmed.

## TRIM21 Structure

**Figure 1:**
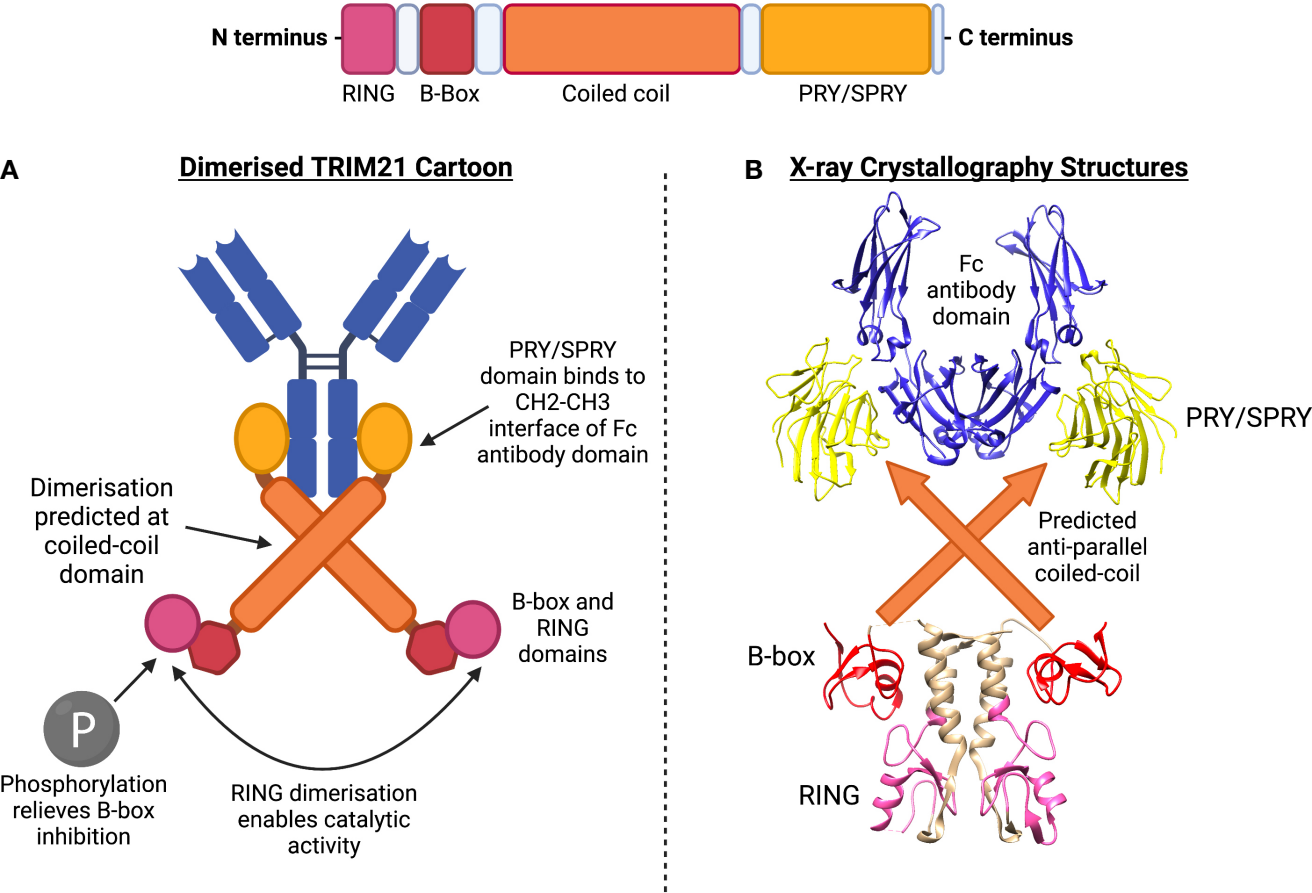
TRIM21 structure includes RING, B-box, coiled-coil and PRY/SPRY domains. **(A)** Dimerised TRIM21 cartoon shows binding of PRY/SPRY domain at constant region (Fc) of antibody. Dimerisation at the coiled-coil is predicted according to homology to other TRIM family members. Phosphorylation of the LxxIS motif of the RING domain relieves B-box inhibition, allowing RING dimerisation for catalytic activity. **(B)** X-ray crystallography structures have been obtained for the (1) PRY/SPRY domain, identified in complex with the Fc antibody domain (PDB:2IWG) (25). (2) The B-box and RING domains have been crystallised in a dimer confirmation (PDB:5OLM) (26). The central predicted coiled-coil structure has not been formally identified.

TRIM21 consists of an N-terminal RING domain, B-box domain, central coiled-coil domain (anticipated based on TRIM family homology) and terminal PRY/SPRY domain (24). The full crystal structure of TRIM21 is yet to be solved. However, TRIM21’s structure can be predicted by combining confirmed structures, binding kinetics and homology. The coiled-coil domain is believed to mediate TRIM21 homodimerization. This enables the dimer’s two PRY/SPRY domains to form a high-affinity binding pocket for immunoglobulin Fc domains, **Figure 1** (25, 27).

### RING Domain

The RING domain is characterised by a series of conserved cysteine and histidine residues (28). These residues coordinate zinc atom binding, enabling the folding of an E3 ubiquitin ligase domain. The E3 zinc finger motifs mediate interactions with ubiquitin-bound E2 enzymes, catalysing the transfer of ubiquitin to target proteins (29, 30). Ubiquitin transfer involves the formation of lysine-specific, covalent polyubiquitin chains, which determine specific cellular activities. For example, proteins modified with Lysine 48 (K48)-specific ubiquitin chains are targeted for proteasomal degradation, whilst K63-specific chains regulate signalling pathways including DNA repair and inflammatory signalling (31).

A specific mechanism for self-anchored TRIM21 ubiquitin transfer has been recently uncovered, showing the RING domain acts as both substrate and catalyst for ubiquitination. Following mono-ubiquitination at the N-terminus (RING domain) by the E2 enzyme Ube2W, TRIM21 is activated. This ubiquitin-priming promotes recruitment of an E2 Ube2N/Ube2V2 heterodimer during K63-specific ubiquitination. Two dimerised RING domains hold the E2-Ub heterodimer in place, catalysing the transfer of K63-linked ubiquitin chains to a third acceptor mono-ubiquitinated RING, during self-anchored *trans*-ubiquitination. Such K63-linked ubiquitination is required for targeting the TRIM21-protein (e.g. antibody) complex for degradation (32).

The RING-domain of TRIM21 also mediates K48-linked ubiquitination. This was demonstrated by the ubiquitination and degradation of DEAD-box protein DDX41, an intracellular dsDNA sensor in myeloid dendritic cells (DCs) and monocytes (33). Binding to DNA *via* the DEAD domain, DDX41 activates the STING pathway for proinflammatory IFN-I stimulation (34). By targeting DDX41 for degradation, TRIM21-mediated DDX41 ubiquitination inhibits the IFN-I response to dsDNA. IFN-I responses to self-DNA are central in SLE pathogenesis (35). It is seemingly contradictory that such IFN-I responses are elevated in SLE patients when TRIM21 mRNA expression is also increased, as one would expect greater DDX41 ubiquitin-mediated degradation (16). This discrepancy may suggest inherent TRIM21 functions in downregulating DNA sensors such as DDX41 are impaired, by some yet unknown mechanism.

### B-Box Domain

The B-box domain is less well-characterised but may have distinct functions in different TRIM family members. It may help coordinate TRIM self-association, may contain a zinc finger motif, in some TRIM proteins might confer E3 ubiquitin ligase activity, or in others has a regulatory role (26, 36, 37).

The B-box domain can mediate higher-order complex formation (38). This was demonstrated for TRIM5α which recognizes and binds to capsids of multiple retroviruses including HIV-1 (39). TRIM5α spontaneously assembles into a hexagonal lattice *via* hydrophobic interactions utilizing a key Arg residue located at the B-box2 domain (40). This hexagonal array mimics that of the target viral capsids and enables efficient binding of the SPRY domains to multiple capsid sites for subsequent ubiquitination and degradation during antiretroviral defense (39).

Despite also having a B-box2 domain, TRIM21 does not form higher-order assemblies. Instead, TRIM21’s B-box domain has a regulatory role *via* interactions with the RING domain (41). Whilst most TRIM proteins are constitutively active, X-ray crystallography has shown that the TRIM21 B-box domain represses its ubiquitin ligase activity by occupying the E2 binding site. Overexpression of kinases IKKβ or TBK1 led to phosphorylation of a LxxIS motif in the RING domain and was sufficient to relieve B-box inhibition (26). These findings may be of interest in an autoimmunity context, as disruption of this autoinhibition mechanism could contribute to excessive proinflammatory signalling. Polymorphisms located in regions encoding the LxxIS motif of TRIM21 have not yet been identified and in general, GWAS studies relating to TRIM21 in autoimmunity are limited. However, one SS patient study identified polymorphisms in multiple coding regions, with certain SNPs correlating with the presence of anti-TRIM21 antibodies (42). Therefore, GWAS studies should be expanded to identify additional polymorphisms associated with TRIM21, including those potentially encoding the LxxIS motif.

### Coiled-Coil

The central coiled-coil domain of TRIM21 is predicted to be an α-helical, supercoiled structure involved in dimerisation and TRIM self-association, **Figure 1** (43). Except for TRIM19 which forms a torus-shaped homo-tetramer, TRIM proteins which associate *via* coiled-coil domains exist either as monomers, homodimers or in monomer: homodimer equilibria, with hetero-dimerisation being uncommon (44, 45). TRIM homodimers (and possibly higher-order oligomers) formed by anti-parallel coiled-coils also allow dimerisation at the RING domain (46, 47).

Studies have shown dimerisation of the catalytic RING domains to be necessary for ubiquitinating activity in most TRIM proteins studied so far (48). This holds true for TRIM21, with a recent study demonstrating that dimerisation at TRIM21’s coiled-coil directly enabled catalytic activity (49). Mutation of residues at the antiparallel coiled-coil dimer interface was sufficient to inhibit auto-ubiquitination of the RING domain, following infection of HEK293T cells with antibody-coated adenovirus. Furthermore, forced RING domain dimerisation (*via* RING-linker-RING constructs) increased ubiquitin discharge activity. A proposed model of “clustering-induced activation” suggests dimerisation of RING domains makes E2-ubiquitin engagement with TRIM21 and subsequent polyubiquitin discharge more energetically favourable (49).

### C-Terminal PRY/SPRY Domain

The variable C-terminal region of TRIM family proteins is involved in protein-protein interaction and subcellular localisation, with TRIM21 containing a PRY/SPRY C-terminal domain, **Figure 1** (25, 45).

The specificity and roles of PRY/SPRY domains have been demonstrated in a number of studies (50). Domain-swapping experiments show that TRIM5α’s PRY/SPRY domain is essential for viral restriction (51). Mutations in TRIM18, most often in the PRY/SPRY domain, alter TRIM18’s subcellular distribution and cause X-linked Opitz Syndrome (52, 53). Furthermore, PRY/SPRY mutations in TRIM20 cause Familial Mediterranean Fever (54), and in TRIM36 cause anencephaly (55).

For TRIM21, the PRY/SPRY domain contains a high-affinity immunoglobulin Fc binding site, the structure of which has been solved, **Figure 1** (25). TRIM21’s PRY/SPRY domain binds to the CH2-CH3 interface of the Fc, and does not overlap with the Fc domain’s binding sites for FcγR and C1q. The CH2-CH3 interface is highly conserved, allowing TRIM21 binding to 98% of circulating immunoglobulins regardless of their antigen specificity (25).

Human TRIM21 displays promiscuous antibody interactions, binding to all four human IgG subclasses and even binding IgGs from different mammalian species in a 2:1 binding ratio (TRIM21: antibody Fc) (56). TRIM21 also binds both IgA (Kd: 54 μM) and IgM (Kd: 17 μM) (57, 58). These affinities are substantially lower than that of IgG binding, which has been measured at Kd: 37 nM for TRIM21-Fc fragment interactions (25). Importantly, except for IgG binding to its cognate high-affinity FcγRI (Kd: 4.2 nm), most FcRs bind IgG Fc with lower-affinity binding in the micromolar range (59, 60). Therefore, the nanomolar TRIM21-Fc binding highlights the strength and high affinity of IgG for this intracellular receptor. Whether TRIM21 can bind to IgE or IgD remains to be investigated (24, 25).

The TRIM21 PRY/SPRY domain is also subject to regulation by acetylation. Histone deacetylase 6 (HDAC6) interacts with TRIM21 *via* the PRY/SPRY domain, where it deacetylases TRIM21 at K385 and K387, promoting TRIM21 homodimerization. When HDAC6 is inhibited, TRIM21 remains hyperacetylated, preventing TRIM21 dimerisation, thus impairing its enzymatic activity (61).

## TRIM21 Anti-Pathogen Activity

Antibodies extracellularly neutralise antigens, preventing targeted pathogens from penetrating cell membranes. This occurs either directly or indirectly through opsonisation and/or complement system activation, whilst antibody-mediated neutralisation within intracellular compartments was thought unlikely due to membrane exclusion (62). Extracellular neutralisation is not always possible, as many neutralising epitopes are shielded, for example by glycans (63).

*In vivo* studies have demonstrated that even in the presence of saturating concentrations of neutralising antibody, TRIM21-deficient mice are highly susceptible to mouse adenovirus 1 infection. In contrast, wild-type mice upregulate TRIM21, thus controlling viraemia. Interestingly, *Trim21^+/-^* heterozygous mice display an intermediate phenotype, leading to an increased viral load but lower than that of *Trim21^-/-^* mice, suggesting that TRIM21 levels directly influence the efficiency of antiviral defence (64).

### Intracellular Antibody Receptor

When bound to non-neutralising antibodies, intracellular pathogens including viruses, bacteria and parasites, may be rapidly sensed by cytosolic TRIM21 (64–67). The mechanisms by which antibody-bound pathogens access the cytoplasm vary and are pathogen-specific (68). Some enveloped viruses, such as the α-herpesvirus HSV, deliver their capsids directly to the cytosol by fusing their envelope with the plasma membrane (69). Others use endocytosis pathways such as clathrin-mediated endocytosis, micropinocytosis or lipid raft-mediated endocytosis (70). Multiple bacterial species use caveolin-mediated endocytosis to enter cells, including *E. coli*, *C. jejuni*, *S. typhimurium*, and *P. aeruginosa*, as have a number of viruses, such as SV40 (71). This mechanism might be preferred for pathogens, as unlike clathrin-coated pit entry, caveolae-internalized bacteria may avoid lysosomal degradation (72).

TRIM21 binds internalised antibody-coated pathogens within the cell *via* PRY/SPRY-Fc interactions. However, why and how antibody-coated pathogens are available for binding in the cytosol, remains largely unsolved. TRIM21 recruitment may be limited to non-enveloped DNA and RNA-viruses that enter the cytosol with attached immunoglobulins. McEwan et al. showed that the enveloped Respiratory Syncytial virus shed attached antibodies upon entry and did not activate TRIM21, whereas non-enveloped feline calicivirus did (73). Additionally, TRIM21 can detect picornavirus HRV14, which promotes the lysis of endosomal membranes for cytosolic virion release, whilst it cannot not detect HRV2, which delivers its genome into the cytoplasm through a pore (74).

**Figure 2:**
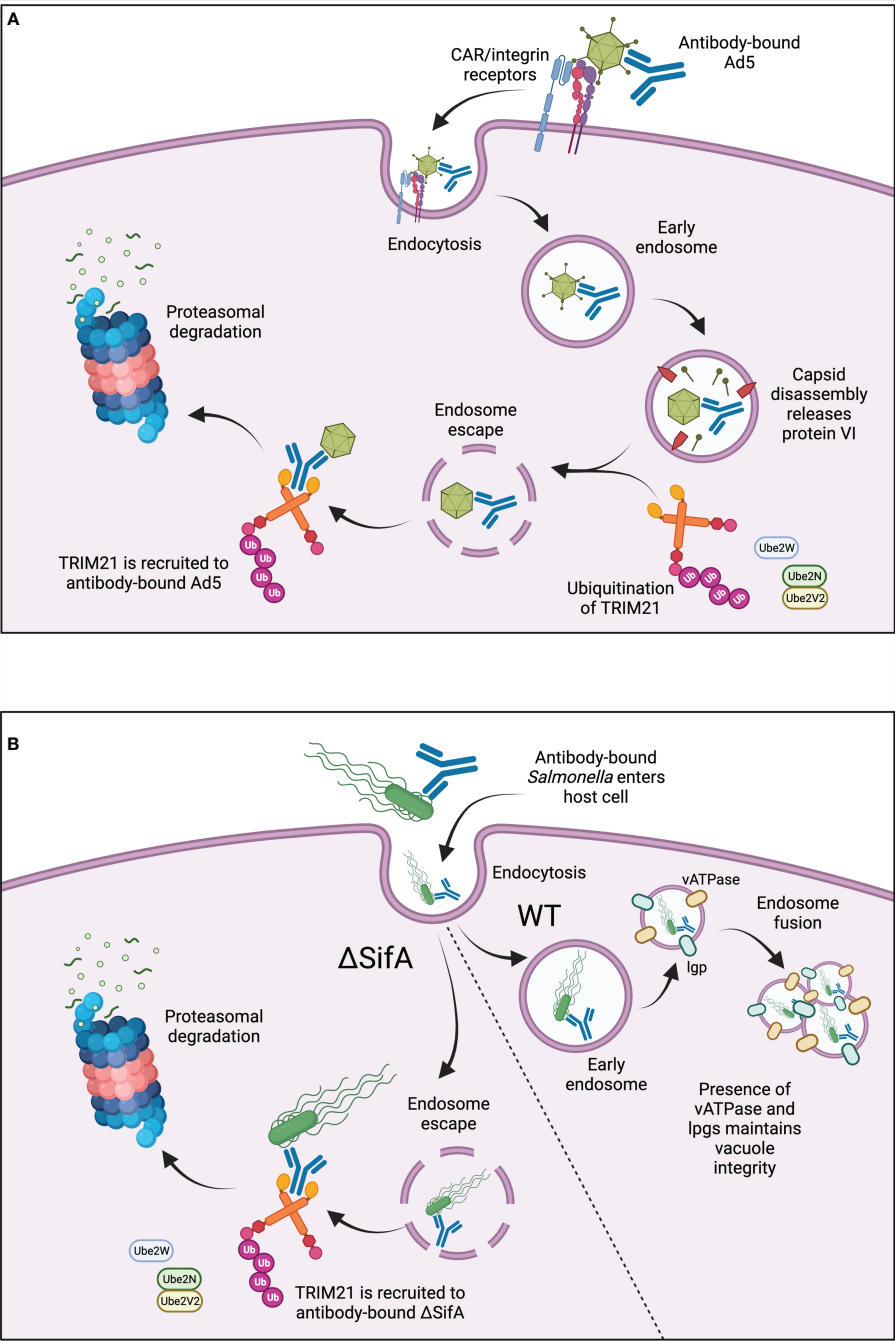
**(A)** TRIM21 in antiviral responses. TRIM21 is recruited to cytosolic antibody-bound-Ad5 after virus entry into the cell. Ad5 binds to CAR/αv integrin receptors at the cell surface, triggering capsid disassembly upon cell entry. Lytic protein VI is released leading to endosomal disassembly and “endosomal escape” of the antibody-bound-Ad5. Active, ubiquitinated TRIM21 binds to the Fc region of the antibody and promotes subsequent proteasomal degradation. **(B)** TRIM21 in responses to intracellular bacteria. TRIM21 is recruited to antibody-coated SifA-mutant *S. typhimurium*. Wild-type *S. typhimurium* are protected from TRIM21-mediated degradation due to the presence of vATPase and lgps, which maintain SCV integrity and enable endosome fusion for bacterial replication. ΔSifA bacteria cannot maintain SCV integrity, leading to endosomal escape into the cytosol for TRIM21-mediated degradation.

TRIM21 facilitates antibody-dependent intracellular neutralisation of human adenovirus type 5 (Ad5). Ad5 bound to as few as 1.6 antibody molecules recruits TRIM21 in the cytosol and is degraded in a TRIM21-dependent manner. This involves TRIM21 autoubiquitination and possibly ubiquitin transfer also to the TRIM21-associated viral particle prior to proteasomal degradation, **Figure 2** (58, 67). Adenoviral capsids can be released from early endosomes into the cytosol during “endosomal escape” (75). This stepwise endosomal rupture process is promoted after initial engagement of Ad5 with coxsackievirus adenovirus receptor (CAR) and coreceptor αv integrin at the host cell membrane (76). Interaction with these receptors may promote early capsid disassembly, occurring just as Ad5 enters the cell membrane (77, 78). Disassembly leads to the exposure of protein VI, a lytic factor which disrupts the endosomal membrane, releasing the capsid into the cytosol for subsequent TRIM21 engagement, **Figure 2** (58, 79).

TRIM21 also localises with antibody-bound *S. typhimurium* in the cytosol, **Figure 2** (73). Normally within host cells, *S. typhimurium* grows within Salmonella-containing vacuoles (SCVs) which protect the bacteria from antibacterial responses (80). However, mutant *S. typhimurium* defective in SifA, an effector translocated by type III secretion systems, have been identified. ΔSifA *S. typhimurium* cannot maintain SCV membrane integrity due to an absence of lysosomal membrane glycoproteins (lgps) and vacuolar ATPase (vATPase), necessary for membrane fusion during bacterial replication. Therefore, ΔSifA mutants are released into the cytosol, **Figure 2** (81). Surprisingly, a follow-up study showed that ΔSifA *S. typhimurium* actually replicated more rapidly within the cytosol of epithelial cells compared to those residing within vacuoles, suggesting there may be some bacterial advantage for vacuole-escape (82). TRIM21 has been shown to recognise antibody-bound-ΔSifA mutants more readily than wild-type *S. typhimurium*, leading to greater activation of NF-κB proinflammatory signalling (73). Although the bactericidal effects of this recognition and enhanced signalling were not explored, it does suggest TRIM21 is important for rapid intracellular detection and binding of pathogens which escape into the cytosol, **Figure 2**.

By binding IgM, TRIM21 provides protection from primary infections, and by binding IgG it can protect against secondary infection. *In vivo* challenge protection has been demonstrated using antibodies targeting the viral nucleoprotein of various viruses including influenza, coronaviruses, and lymphocytic choriomeningitis virus (LCMV) (65, 83–89). Nucleoprotein-specific antibodies are non-neutralising, as viral nucleoprotein is an internal virus antigen, and protection does not require the FcγR (90, 91). TRIM21 and anti-nucleoprotein antibodies play a critical role in stimulating nucleoprotein-specific cytotoxic T cells during LCMV infection (65).

Once bound to cytosolic antibody-coated pathogens, TRIM21 induces coordinated effector and signalling responses, both dependent on TRIM21’s E3 ubiquitin ligase activity. Upon cellular infection with Ad5-antibody complexes, two independent, concurrent activities occur. Firstly, the effector response results in proteasomal degradation of the virus. Secondly, an intracellular antiviral response is induced *via* innate-immune signalling molecules such as IFN-I (67, 68). Additionally, TRIM21 initiates signalling cascades, activating the transcription factor NF-κB, upregulating more inflammatory cytokines and inducing an antiviral state (73).

### Viral Subversion of TRIM21

Some viruses have hijacked TRIM21 to enhance their replication. In the case of severe fever with thrombocytopenia syndrome virus, its non-structural protein (NSs) interacts with and inhibits TRIM21. This prevents the activation of nuclear factor erythroid 2-related factor 2, which is responsible for the expression of a number of antioxidant effectors, thus promoting viral replication and pathogenesis (92). Enterovirus 71 (EV71) is restricted by the host restriction factor SAMHD1. Upon infection, EV71 upregulates TRIM21 in an IFN-dependent manner; TRIM21 interacts with and degrades SAMHD1 through K48-ubiquitination and proteasomal degradation, thus promoting EV71 replication (93). Human papillomavirus presents an alternative method for immune escape by utilising the oncoprotein E7. This recruits TRIM21 to promote the K33-linked ubiquitination and degradation of the host IFI16 inflammasome (94).

### Other Interactions

TRIM21 also has indirect effects on innate immunity by promoting pattern recognition receptor (PRR) detection of exposed immunostimulatory ligands that are otherwise shielded by viral capsids. This induces two waves of TRIM21-dependent transcription, the first caused by TRIM21 antigen recognition *via* Fc binding and a later wave following recognition of viral PAMPs by cGAS and STING (74). TRIM21 also interacts through its PRY/SPRY domain with another PRR, MAVS. TRIM21 is upregulated following early detection of host cell viral invasion from Hepatitis C virus, Newcastle disease virus or Sendai virus (SEV). Then its RING domain conjugates K27-linked polyubiquitin onto MAVS, promoting TBK1 binding for subsequent MAVS downstream signalling (95, 96).

Effects of TRIM21 on virus-encoded proteins are less well-understood than those of the IFN pathway. TRIM21 reportedly interacts with hepatitis B virus (HBV) to prevent HBV DNA replication, by promoting the K48-linked ubiquitination and degradation of HBV DNA Pol (97). Additionally, Porcine Epidemic Diarrhoea Virus (PEDV) is inhibited by TRIM21, which targets the nucleoprotein for proteasomal degradation. Conversely, following *in vitro* PEDV infection, the endogenous expression of TRIM21 was downregulated, increasing both PEDV viral titres and nucleoprotein levels (98).

Co-immunoprecipitation has shown TRIM21 to interact with intracellular proteins that regulate antiviral responses. This includes direct interactions with IFN-inducible protein 35 (IFI35) and indirectly, the N-Myc and STAT interactor (NMI). Subsequent K63-linked ubiquitination of NMI stabilises the TRIM21/IFI35/NMI complex and downregulates innate antiviral signalling pathways by inhibiting IFN-I production (99).

## TRIM21 – An Interferon Stimulated Gene

### Expression

**Figure 3:**
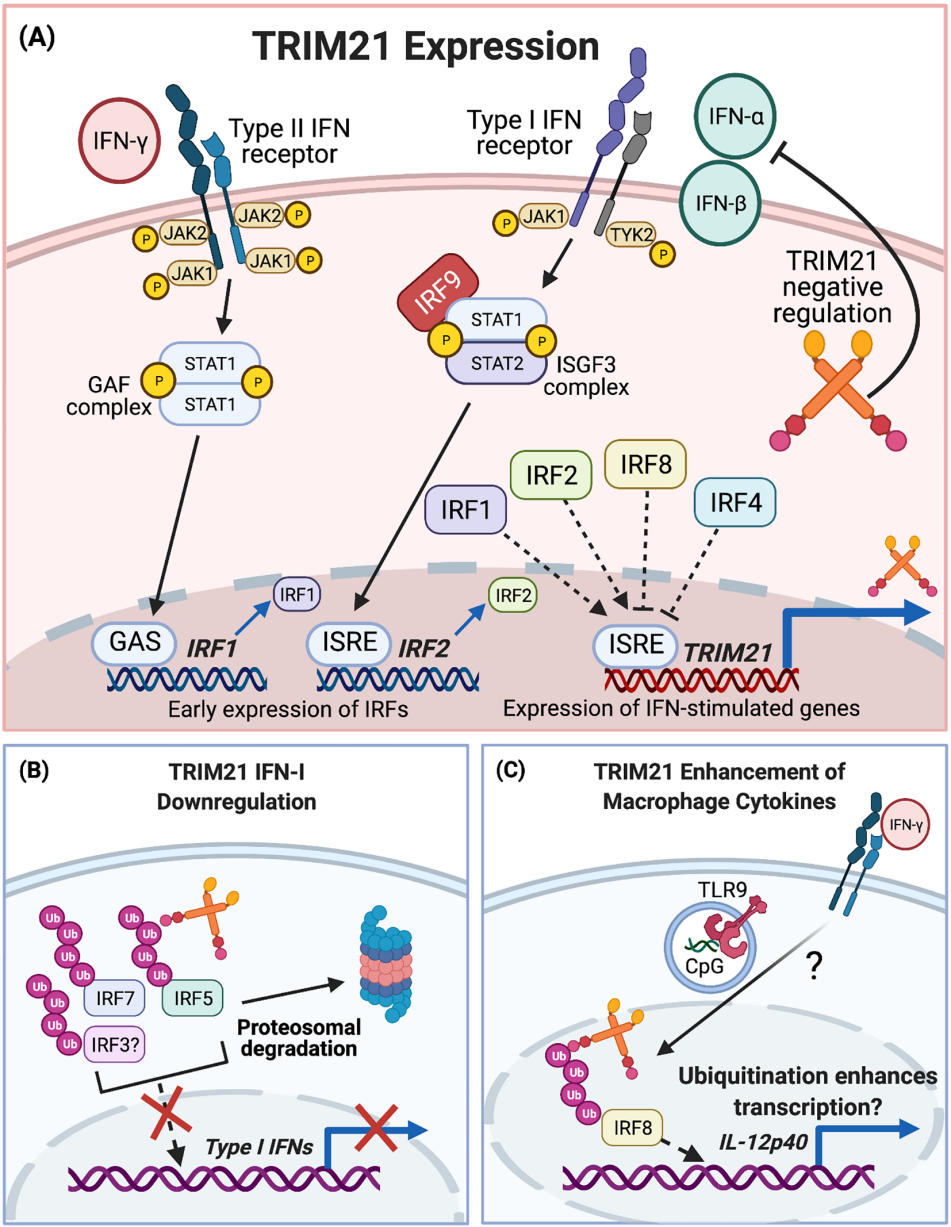
TRIM21 and IFN signalling. **(A)** TRIM21 expression is upregulated by IFN-I and IFN-II signalling. IFN-I signalling involves JAK/STAT phosphorylation and ISGF3 complex formation for early IRF expression. Nuclear translocation of IRF1/2 and binding to the ISRE leads to expression of IFN-stimulated genes including TRIM21. IFN-II signalling also occurs *via* JAK/STAT signalling, leading to formation of the GAF complex. This binds to GAS elements for IRF1 expression and subsequent TRIM21 expression. TRIM21 upregulation is inhibited by IRF4 and IRF8, although much of the upstream signalling pathway remains to be fully elucidated. **(B)** After upregulation, TRIM21 downregulates the IFN-I response *via* ubiquitination of IRF7, IRF5 and possibly IRF3. **(C)** TRIM21 enhances proinflammatory macrophage cytokine expression after IRF8 ubiquitination.

IFNs bind to cell surface receptors, triggering signalling cascades for downstream transcription of genes involved in innate and adaptive immunity. IFN-α and IFN-β are type I IFNs (IFN-I) and IFN-γ is type II (IFN-II) (100). Early indications that TRIM21 is regulated by IFN signalling were shown by Rhodes et al. In unstimulated HeLa cells, TRIM21 was detected at low concentrations, whilst TRIM21 mRNA was rapidly upregulated in response to IFN-II (101). Furthermore, TRIM21 was upregulated in cultured macrophages and DCs, in response to either influenza virus infection or CpG oligonucleotide TLR9 agonists. TRIM21 induction was dependent on IFN-I signalling, as it was diminished in IFNAR^-/-^ cells (102). IFN-I and to a lesser extent IFN-II, induced TRIM21 mRNA in the mouse T cell line EL4. Promoter-mapping and luciferase-reporter experiments identified an IFN-stimulated response element (ISRE) in the TRIM21 promoter, **Figure 3** (103).

IFN-I stimulation induces phosphorylation of the JAK1 and TYK2 kinases, which in turn phosphorylate STAT1 and STAT2. pSTAT1 and pSTAT2 complex with IRF9, forming the IFN-stimulated gene factor 3 (ISGF3) complex which translocates to the nucleus. ISGF3 activates transcription by binding ISREs in promoters of IFN-stimulated genes, **Figure 3** (104). IFN-II signalling induces JAK1 and JAK2 phosphorylation, leading to STAT1 phosphorylation and the formation of pSTAT1 homodimers, referred to as the gamma-activated factor (GAF) complex. GAF translocates to the nucleus, binding to gamma IFN activation sites (GAS) to activate transcription, **Figure 3** (105).

Both IRF1 and IRF2 bind to a *TRIM21* gene ISRE to upregulate TRIM21 expression, **Figure 3** (103, 106). Despite the abrogation of IFN-II-stimulated TRIM21 induction in IRF1^-/-^ macrophages, the TRIM21 locus does not contain a GAS site. This suggests IFN-II stimulates TRIM21 expression by an indirect mechanism involving IRF1. TRIM21 expression was inhibited by IRF4 and IRF8, which bound to the ISRE and blocked IRF1 and IRF2-mediated TRIM21 upregulation, **Figure 3** (103). The roles of non-canonical IFN signal transduction pathways in *TRIM21* gene regulation are not yet known (107).

### Negative Regulation

TRIM21 participates in a negative feedback loop to downregulate IFN-I production, modulating the IFN response and inhibiting further TRIM21 expression, **Figure 3** (108, 109). Downregulation of IFN-I results from TRIM21-mediated IRF7 ubiquitination and subsequent proteasomal degradation, **Figure 3**. In TRIM21-deficient cells, these inhibitory effects were abrogated and the IFN-α4 promoter was overstimulated by IRF7 (109).

Similarly, TRIM21 ubiquitinates IRF5 to mediate its degradation and prevent IRF5-driven IFN-α expression, **Figure 3** (110, 111). Interestingly, one study showed that although TRIM21 interacted with and ubiquitinated all IRF5 isoforms studied, the subsequent effects on protein stability were isoform specific. For example, IRF5 isoforms lacking 48 nucleotides in a central Proline, Glutamic acid, Serine, Threonine-rich (PEST) domain were ubiquitinated by TRIM21 but resistant to subsequent degradation (110). Increased stability of certain isoforms may have relevance in SLE pathogenesis, whereby patients show enhanced expression and alternative splicing of IRF5, due to increased spliceosome activity (112). IRF5 polymorphisms affecting PEST domain expression are associated with elevated risk for SLE and may modulate IFN-I promoter activity. However, it remains unclear whether these isoforms contribute to SLE pathology through increased IFN-I expression (113).

Evidence for IFN-β downregulation following TRIM21-mediated IRF3 degradation, is more conflicting. In a human glial cell line (CHE3), Japanese encephalitis virus infection stimulated IFN-β upregulation *via* IRF3 phosphorylation. TRIM21 overexpression abrogated IFN-β induction, whilst TRIM21 silencing enhanced it (108). These findings agreed with a previous study in HEK293 cells, whereby IRF3-induced IFN-β expression was negatively regulated by TRIM21 following SEV infection, or stimulation by LPS or the TLR3 agonist polyI:C, **Figure 3** (114). In contrast, after SEV infection of HEK293 cells, TRIM21 bound IRF3, blocking Pin1-mediated pIRF3 degradation and enhancing IRF3-stimulated IFN-β expression (115, 116). Reasons for these conflicting results remain unknown and the impact of TRIM21-mediated ubiquitination on the stability of IRF3 is uncertain.

### Nuclear Translocation

Studies have also investigated IFN-mediated upregulation of TRIM21 and the downstream effects leading to apoptosis and macrophage cytokine expression (117, 118). Imaging using fluorescent anti-TRIM21 monoclonal antibodies showed that IFN-I stimulation of HeLa cells led to increased cytoplasmic TRIM21 protein levels. After 48 hours of stimulation TRIM21 had undergone nuclear translocation which preceded apoptosis, detected by TUNEL-assays in parallel-treated cultures (117). However, whether TRIM21 nuclear translocation actively induced apoptosis remains to be confirmed. The mechanisms of nuclear translocation, and the functions, if any, of intranuclear TRIM21 are yet to be explored.

Intranuclear roles of TRIM21 have been demonstrated in macrophages following IFN-II and TLR stimulation. IRF8-TRIM21 complexes were identified in nuclear extracts of macrophages stimulated with IFN-II plus TLR9 agonist CpG oligonucleotides (118, 119). This was verified in live macrophages whereby YFP fluorescent signals dependent on IRF8-TRIM21 interactions were detected in the nucleus, also corresponding to increased IRF8 ubiquitination. However, unlike previous reports, this led to enhanced proinflammatory IL-12p40 expression, suggesting that ubiquitination actually enhances IRF8-mediated transcription, **Figure 3** (118). Much of the upstream signalling pathway and how IRF8-ubiquitination enhances IL-12p40 transcription remains unclear. It may be worth confirming whether similar examples of enhanced TRIM21-mediated transcription exist for other proinflammatory gene targets in activated macrophages. These could include promotors requiring coordinated regulation by both IRF8 and IRF1, including SOCS7 for NF-κB signalling, H28 for antigen presentation, c-Myc for cell growth and survival, and CXCL16, which is elevated in SLE patient sera and expressed by macrophages for adhesion, chemotaxis and inducing inflammatory cell infiltration (120, 121).

### Importance of Regulation

Inducing IFN-I signalling helps eliminate virus and prevents host morbidity and mortality (122–124). However, IFN levels must be balanced; too much may over-stimulate innate immunity and inflammation, leading to pathological outcomes including autoimmunity. Therefore, signal termination is necessary to prevent an excessive IFN response. TRIM21 controls innate antiviral responses by degrading the transcription factors IRF7 and IRF3 (108, 125, 126). For Influenza A virus, NMI is upregulated, promoting TRIM21 ubiquitination of IRF7 (125), whilst for SEV infection, FoxO1 destabilises IRF3 by promoting TRIM22 or TRIM21-mediated proteasomal degradation, following K-48 ubiquitination (126).

Autoimmunity as a result of aberrations in these fine-tuned signalling pathways has been demonstrated for SS using gene expression array analysis. Compared to healthy controls, PBMCs isolated from SS patients displayed elevated TRIM21 transcript expression, correlating with increased IRF1 and IRF2 mRNA (103). One might expect enhanced ubiquitination of TRIM21-regulated IRFs, leading to greater downregulation of the IFN response, yet this was not the case. Instead, IRF3 and IRF5 protein levels remained high even in the presence of increased TRIM21. This may suggest other signalling pathways contribute to impaired proteasomal degradation and altered IFN signalling homeostasis in autoimmune individuals (127). A clearer understanding of these signalling pathways is needed.

## TRIM21 and Dysregulation of Cellular Homeostasis

### In Cancer

TRIM21 downregulation has been implicated in unfavourable outcomes for multiple cancer types including breast cancer, hepatocellular carcinoma, diffuse large B-cell lymphomas (DLBCL) and most recently, colitis-associated cancers (128–131). Lower TRIM21 mRNA expression was correlated with reduced patient survival in DLBCLs and in hepatocellular carcinomas; *in vitro* studies implicated enhanced cell proliferation as a possible cause (129, 130). In liver cancer cell lines, siRNA-mediated TRIM21 silencing abrogated apoptosis, whilst promoting cellular proliferation and migration in a transwell system (129). Conversely, TRIM21 overexpression in a B cell line reduced proliferation and increased apoptosis compared to control cells, and promoted apoptosis following anti-CD40 treatment (16). Together, these studies suggest TRIM21 has tumour suppressor capabilities, influencing cellular and tissue homeostasis.

Autoantibodies against nuclear and cytoplasmic autoantigens have been detected in cancer patients, with possible causal mechanisms including autoantigen overexpression from tumour cells and autoantigen release following chemotherapy-induced cell death (132). Recently, anti-TRIM21 antibodies have been detected in patients with ovarian cancer, the presence of which correlated with better patient survival. Analysis showed these antibodies differed in epitope profile to anti-TRIM21 antibodies detected in SLE and SS, although why this is the case remains to be discovered (133).

### TRIM21 Deficiency and Autoimmune B Cells

Dysregulation of B cell function has been identified in both SS and SLE patients. This is characterised by higher levels of circulating activated B cells which express co-stimulatory markers and secrete autoantibodies, driving inflammation and autoimmune pathology (2). Similarly to the cancer studies, TRIM21 deficiency has been explored as a potential driver of SLE, by altering B cell regulation. TRIM21^-/-^ mice have been developed with differing phenotypes, most likely due to specific gene disruption strategies (134). One knockout showed no phenotypic abnormalities, likely due to compensatory upregulation of related TRIMs (135). However, other TRIM21^-/-^ mice showed inflammatory phenotypes when subjected to minor injury by ear tagging. They developed site-specific severe dermatitis and displayed SLE-specific autoimmune characteristics, including elevated proinflammatory cytokine production (136). This discrepancy may occur due to production of a truncated TRIM21 protein (RING, B-box and partial coiled-coil domain) in the second strategy, which enabled and promoted autoantibody production.

**Figure 4:**
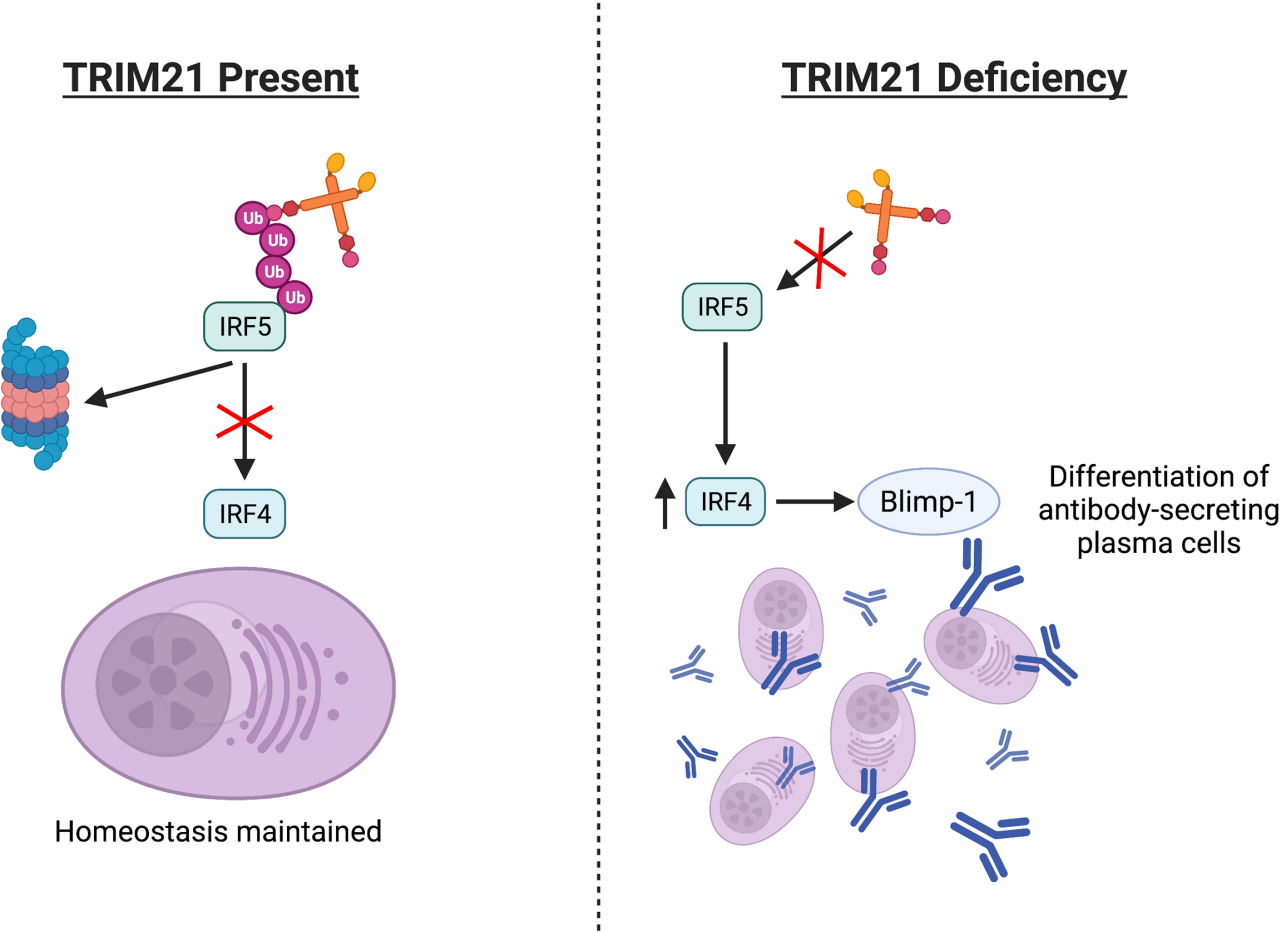
Absence or deficiencies in TRIM21 may alter the IRF4/5 axis. TRIM21 regulates IRF5 *via* ubiquitination, potentially affecting downstream IRF4 and Blimp-1 expression. Aberrant regulation may increase differentiation of antibody-secreting plasma cells.

Another study showed how *TRIM21* gene disruption in a lupus-prone mouse model (TRIM21^−/−^MRL/*lpr*) altered SLE pathology. Compared to lupus-prone (MRL/*lpr)* mice retaining functional TRIM21 activity, the spleens of TRIM21^−/−^MRL/*lpr* mice had significantly increased mature B cell numbers. Once isolated, these cells differentiated into antibody-secreting plasma cells at higher rates *in vitro*, compared to cells from the MRL/*lpr* spleens (137). One model proposed TRIM21 may regulate plasma cell differentiation by ubiquitinating IRF5, thus blocking IRF5-induced upregulation of IRF4 and subsequent Blimp-1 mRNA expression, **Figure 4** (110, 137–139). However, much of this pathway, especially how IRF5 alters IRF4 expression and whether TRIM21 has a definitive role in this IRF4/5 axis, requires clarification.

### Impaired Function

Alternatively to expression deficiencies, impaired TRIM21 function may involve antibody-dependent mechanisms. For example, anti-TRIM21 seropositivity in SLE patients correlates with impaired TRIM21 ubiquitinylating activity. PBMCs isolated from anti-TRIM21 seropositive patients display enhanced IFN-I gene expression, suggesting an association between the presence of TRIM21 autoantibodies and attenuated negative regulation of IFN-pathway proteins by TRIM21 (127).

How autoantibodies may downregulate native TRIM21 function has been explored *in vitro*. In a cell-free system, anti-TRIM21 antibodies from SS patients sterically hindered TRIM21’s interaction with the E2 ubiquitin-conjugating enzyme, preventing E3 ligase autoubiquitination *via* the RING domain (140). This may impair the subsequent ubiquitination of target proteins such as IRF5, although whether anti-TRIM21 antibodies can indeed access and inhibit TRIM21 *in vivo* has not been directly tested.

## How Can Antibodies Access Intracellular TRIM21?

Under normal conditions, TRIM21 is uniformly distributed throughout the cytoplasm, with small amounts detected within the nucleus (141). Thus, whether and how autoantibodies access this cytoplasmic protein remains a key question for TRIM21-associated autoimmune diseases.

### Cell Surface Expression

Recently, TRIM21 was detected at the surface of antigen-presenting cells in pSS patient blood (142). Anti-TRIM21 antibodies are associated with pSS pathogenesis, inducing damage and driving inflammation. This could involve recognition of anti-TRIM21 Fc domains by lymphocytes, driving inflammatory pathways such as antibody-dependent cellular cytotoxicity (143, 144). Therefore, cell surface TRIM21 might exacerbate proinflammatory pathways in autoimmune diseases.

IFN-β, the most potent inducer of TRIM21 expression, induces changes in TRIM21 expression and cellular location in PBMCs (142, 145). TRIM21 was strongly upregulated both intracellularly and at monocyte cell surfaces, with a modest increase observed in plasmacytoid DCs (142). Although this evidence suggests IFN upregulates TRIM21 expression, it remains unclear how TRIM21 may be transported and expressed at the cell surface.

### Exposure After Apoptosis

**Figure 5:**
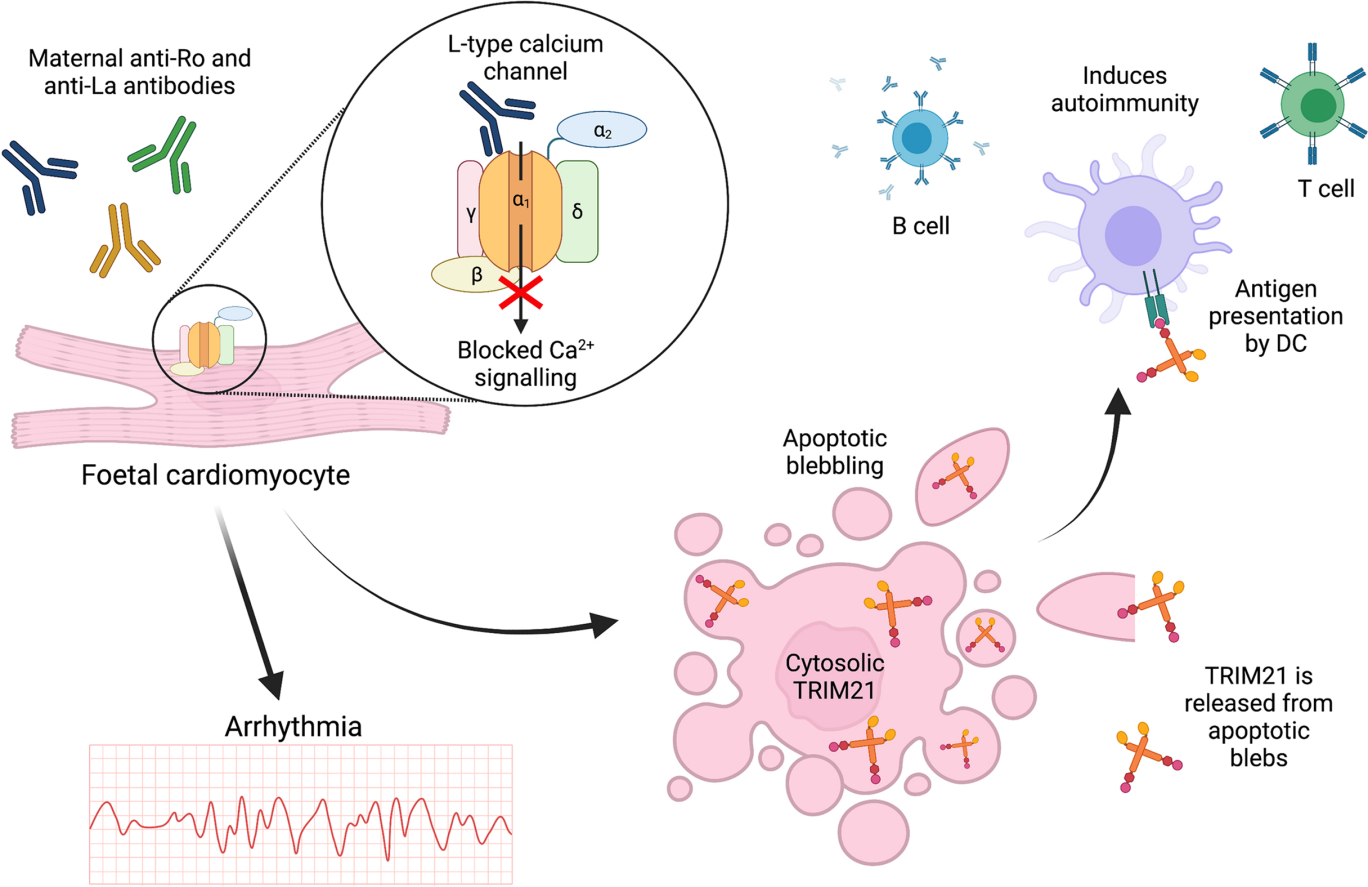
TRIM21 exposure at the cell surface may occur due to apoptosis. In foetal CHB, cross-reactive maternal autoantibodies may bind to and block L-type calcium channels leading to arrhythmia and cardiomyocyte apoptosis. This releases intracellular antigens such as TRIM21 for antigen presentation, driving subsequent autoimmunity.

These recent novel findings contradict previous publications suggesting TRIM21 is only exposed at the cell surface during cell death, with anti-TRIM21 antibodies possibly driving apoptosis (141–143). TRIM21 exposure following apoptosis has been demonstrated in the context of foetal CHB which is strongly associated with maternal SLE and SS (146). High titres of cross-reactive maternal anti-TRIM21 antibodies induced apoptosis of foetal cardiomyocytes *in vitro*, **Figure 5** (9). Although CHB pathogenesis remains unclear, one proposed mechanism involves cross-reactive, inhibitory binding of maternal anti-Ro52/60 and anti-La antibodies to α1-subunits of L-Type foetal cardiomyocyte calcium channels (147). Channel inhibition may then dysregulate calcium signalling, leading to abnormal atrioventricular conduction and eventual apoptosis, **Figure 5** (148, 149). Apoptosis of cultured, non-permeabilised human foetal cardiomyocytes led to the redistribution of Ro52/60 and La antigens into apoptotic blebs and their emergence at the cell surface (150). Furthermore, in a co-culture system, apoptotic foetal cardiomyocytes treated with anti-Ro/La antibodies stimulated macrophages to secrete TNF-α (151). Apoptotic blebs rich in autoantigens such as TRIM21 may be insufficiently cleared, facilitating antigen-autoantibody interactions, or enabling DC ingestion and presentation to activate T cells and autoreactive B cells, **Figure 5** (152–154).

### Exposure After Stress-Induced Translocation

TRIM21 may also be exposed in non-apoptotic cells following UV-B/C exposure or oxidative stress (155, 156). For example, UV-irradiated keratinocytes displayed TRIM21 at the cell surface, a phenomenon which was inhibited in a concentration-dependent manner by the reactive oxygen species (ROS) scavenger *N*-acetyl-L-cysteine (157, 158). Surface TRIM21 exposure was also induced in keratinocytes treated with diamide, a thiol-oxidising agent (157). Diamide disrupts intracellular glutathione redox reactions, inducing oxidative stress and promoting mitochondrial-induced apoptosis pathways (159). Together, these results indicate that oxidative stress and altered redox homeostasis may induce cell surface TRIM21 exposure. Increased expression of molecular chaperones may also be involved, aiding TRIM21 protein synthesis and translocation, although this has not been confirmed experimentally (157).

Oxidative damage has been identified in the salivary glands of SS patients, with increased saliva detection of the oxidative stress biomarker 8-Hydroxy-2-deoxyguanosine, generated following ROS-induced oxidation of DNA guanine residues (160, 161). The findings by Saegusa et al., suggest that ROS may drive increased exposure of TRIM21 during SS pathogenesis (157). Other studies have confirmed the translocation of SLE autoantigens to keratinocyte cell surfaces following stimulation by UV-B, heat-shock or certain inflammatory cytokines (144). However, the optimal irradiation dose (200mJ/cm^2^) used in one study far exceeds levels required to induce significant keratinocyte cell death (162, 163). Thus, the autoantigen redistribution observed in these studies might have occurred during early apoptotic events.

Whilst the mechanism by which TRIM21 is translocated to the surface remains uncertain, one study reported that TRIM21 cytoplasmic bodies localise along microtubule networks. Visualised using live cell fluorescence microscopy, these bodies were highly mobile, undergoing multidirectional movements across a range of distances within the cytosol. However, motility was only observed beneath the plasma membrane and not at the cell surface (164). Therefore, whether this motility contributes to TRIM21 membrane exposure in response to stimulation or oxidative stress remains unclear.

Together these reports suggest that TRIM21 translocation to the cell surface may occur following apoptosis or cellular stress. Coming back to the recent findings by Hillen et al., it is possible the observed TRIM21 surface expression actually occurred during early apoptotic events (142). Repeating this study using Annexin V as a discriminator between apoptotic and non-apoptotic cells may elucidate whether TRIM21 is indeed expressed at the cell surface under non-apoptotic conditions (165).

## Anti-TRIM21 Antibody Development in Patients and Animal Models

### Role of BAFF in Anti-TRIM21 Responses

Defective tolerance mechanisms, elevated antigen presentation and inflammatory cytokine expression are thought to drive autoreactive B cell activation, leading to pathogenic autoantibody production (166). Therefore, it is noteworthy that TRIM21 protein levels are significantly elevated in the salivary gland ductal epithelium of SS patients, correlating with inflammation and lymphocyte infiltration (167). Studies have directly correlated serum anti-TRIM21 antibody levels with disease severity. Higher anti-TRIM21 autoantibody titres in pSS are associated with greater localised and systemic disease manifestations including parotid enlargement, haematological abnormalities and central nervous system involvement (168). Studies of patients undergoing hematopoietic stem cell transplantation suggest a link between elevated BAFF expression and anti-TRIM21 autoantibodies (169). Increased BAFF may support the survival of TRIM21-targeting autoreactive memory B cells. However, questions remain as to where this tolerance breakdown occurs, whether TRIM21 specifically drives autoreactivity and whether BAFF expression is necessary for anti-TRIM21 responses.

### Epitope Spreading

Another potential mechanism driving anti-TRIM21 autoantibody production is epitope spreading, whereby during chronic inflammation, the exposure of normally sequestered self-antigens induces a secondary immune response against endogenous epitopes (170, 171). Such epitope spreading was demonstrated in mice immunised with the Ro60 autoantigen plus Freund’s complete adjuvant. After repeated Ro60 booster immunizations, mice developed an antibody response against the unrelated TRIM21/Ro52 autoantigen (172). This corresponds with SS patient data whereby individuals may display anti-TRIM21 antibodies alone, or in combination with anti-Ro60 antibodies (173). Interestingly, the autoantibodies from anti-TRIM21/anti-Ro60 doubly-seropositive patients exhibit a distinct epitope profile with frequent reactivity to the TRIM21 RING domain, in contrast to patients reactive to TRIM21 alone (174). Thus, Ro60 autoimmune reactivity might influence subsequent reactivity against TRIM21.

Importantly, TRIM21 and Ro60 are structurally and functionally distinct proteins occupying different cellular compartments. Ro60 (TROVE2) a ring-shaped protein which forms ribonucleoprotein complexes with non-coding (Y) RNA. By binding misfolded Y-RNAs, Ro60 may assist in their degradation (175). Direct interactions and complex formation between Ro60 and TRIM21/Ro52 have not been established, although transient interactions may be possible (175, 176). One hypothesis for how epitope spreading could induce double-positive TRIM21/Ro60 autoreactivity, is that if an autoimmune patient displays reactivity against one of these antigens (Ro60 or TRIM21), transient antigen interactions may expose epitopes from the other antigen, driving a secondary autoimmune response (177). However, questions remain regarding the autoimmune contributions of reactivity against certain TRIM21 epitopes and why only some autoimmune patients are double-positive for anti-Ro60 and anti-TRIM21 antibodies.

### Modelling Anti-TRIM21 Autoimmunity

Autoimmune induction of anti-TRIM21 antibodies has been explored in mouse models (178). In particular, the New Zealand Mixed 2758 (NZM2758) mouse strain, obtained by backcrossing and selectively breeding New Zealand Black and New Zealand White mice, has been used to model SS development (179, 180). These mice display classical SS manifestations including glandular histopathology and dysfunction, lymphocyte infiltration, inflammation and autoantibody production (180). TRIM21-immunized NZM2758 mice developed salivary gland dysfunction which correlated with levels of anti-TRIM21 produced (181). Passive serum transfer from TRIM21-immunized NZM2758 mice induced salivary gland dysfunction in adjuvant-primed (intraperitoneally), non-immunized recipient NZM2758 mice (181).

Of note, TRIM21 immunization induced IgG deposition in, and dysfunction of, the lacrimal glands in female but not male NZM2758 mice (182). This observation is interesting in light of the known sex differences in pSS presentation, with women accounting for approximately 90% of cases but men typically presenting with more severe disease (183). Male mice in the Trzeciak et al., study showed no significant clinical disease and therefore do not model the severe disease observed in men with pSS (182). Sex differences in the pathogenesis of rheumatic diseases such as SS and SLE may be related to hormonal effects (184) and to gene dosage effects of immune-related genes such as TLR7 on the X chromosome (185).

### Anti-TRIM21 Epitope Targets

Immunisation of NZM2758 mice with MBP-fusion proteins corresponding to different murine TRIM21 domains, was used to determine anti-TRIM21 antibody epitope targets and how they relate to autoimmune pathologies. No differences were detected in the levels of autoantibodies generated against the different TRIM21 domains, however certain epitopes correlated with salivary gland dysfunction in these mice. Specifically, antibodies raised against the coiled-coil domain of TRIM21 reduced saliva production, suggesting a link between specific antibody targets and SS pathology (186). This corresponds with patient findings, confirming that the coiled-coil domain is the most antigenic epitope. For example, 97% of patients with autoimmune rheumatic diseases, and who are also anti-TRIM21 antibody positive, generate antibodies targeting the coiled-coil domain, **Figure 1** (187).

Extensive SS-patient epitope analysis has demonstrated the presence of autoantibodies directed against other TRIM21 regions, including the immunoglobulin binding C-terminal (PRY/SPRY) domain, **Figure 1**. Using pull-down assays, it was demonstrated that antibodies from patient serum bound with high affinity to the C-terminal region of both wild-type TRIM21 and a mutant which cannot bind to immunoglobulin Fc. One model proposes that TRIM21’s role in targeting antibody-coated pathogens for proteasomal degradation may promote autoantibody production against these antigenic regions (24, 188). However, whether or not autoantibodies directed against the C-terminal domain are pathogenic and actively drive SS clinical manifestations (e.g. glandular dysfunction), remains to be elucidated.

## Conclusion and Future Perspectives

TRIM21 is an intracellular receptor that binds with high affinity to immunoglobulin Fc regions *via* its PRY/SPRY domains. This binding is vital for intracellular detection of pathogens that escape extracellular antibody neutralisation, enter host cells and access the cytosol. Subsequently, ubiquitination targets the pathogens for proteasomal degradation and immune signalling is activated. In particular, TRIM21 is both enhanced by and regulates IFN signalling for effective immune responses to infection. This also includes the upregulation of other proinflammatory transcription factors such as NF-κB, inducing an antiviral state for pathogen destruction.

TRIM21 dysregulation has been implicated in cancers and autoimmune diseases which include SS and SLE. In particular, excessive IFN responses are correlated with elevated TRIM21 transcript expression. However, these findings are seemingly counter-intuitive as more TRIM21 should promote greater IRF ubiquitination, thus decreasing the IFN response. Therefore, clearer understanding of TRIM21’s roles in these signalling pathways is required to explain these contradictory findings.

The associations between anti-TRIM21 seropositivity and impaired intracellular TRIM21 ubiquitination activities are intriguing. However, the main question these findings raise is how autoantibodies are generated against an intracellular antigen. Whether TRIM21 is expressed at the cell surface remains unclear, whilst exposure after apoptosis, as shown by apoptosis of foetal cardiomyocytes in the presence of maternal anti-Ro52 antibodies, provides the most suitable explanation for the generation of anti-TRIM21 antibodies.

Why only some autoimmune patients generate antibodies against both Ro60 and TRIM21/Ro52, whilst others generate antibodies against just one, is another unanswered question. So far, NZM2758 mice have provided a model of female-specific SS. Further studies using these animal models may answer key questions related to anti-TRIM21 antibody generation, autoantibody epitope specificity, and how this relates to SS clinical manifestations. Certainly, answering these questions will go some way to enhancing our understanding of TRIM21 biology, its roles in innate and adaptive immunity, and its contributions to autoimmunity.

## Author Contributions

EJ, SL, and LD: Researched the topic, wrote, and edited the manuscript. EJ: Prepared the illustrations. All authors contributed to the article and approved the submitted version.

## Funding

EJ is supported by a KTRR studentship from the Kennedy Trust for Rheumatology Research (KENN 19 20 01), and by a Henni Mester Fellowship (University College, University of Oxford). SL was supported by the Kennedy Trust for Rheumatology Research (research grant KENN 18 19 01).

## Conflict of Interest

The authors declare that the research was conducted in the absence of any commercial or financial relationships that could be construed as a potential conflict of interest.

## Publisher’s Note

All claims expressed in this article are solely those of the authors and do not necessarily represent those of their affiliated organizations, or those of the publisher, the editors and the reviewers. Any product that may be evaluated in this article, or claim that may be made by its manufacturer, is not guaranteed or endorsed by the publisher.
